# The Use of the Gliding Arc Plasma Technique to Deposit Fe or Mn Oxides on Fibrous Ceramic Supports for Reactions of Environmental Interest

**DOI:** 10.3390/ma18245479

**Published:** 2025-12-05

**Authors:** Sabrina Antonela Leonardi, Maximiliano Rodriguez, Eduardo Ernesto Miró, Eric M. Gaigneaux, Viviana Guadalupe Milt

**Affiliations:** 1 Facultad de Ingeniería Química, Instituto de Investigaciones en Catálisis y Petroquímica, INCAPE (UNL-CONICET), Santiago del Estero 2829, Santa Fe 3000, Argentina; sleonardi@fiq.unl.edu.ar (S.A.L.); maxir_15@hotmail.com (M.R.); emiro@fiq.unl.edu.ar (E.E.M.); 2 Institute of Condensed Matter and Nanosciences (IMCN), Université Catholique de Louvain (UCLouvain), Place Louis Pasteur 1, 1348 Louvain-la-Neuve, Belgium

**Keywords:** plasma deposition, biomorphic ZrO_2_ fibers, Mn and Fe oxides, CO oxidation, soot removal

## Abstract

The gliding arc plasma technique (glidarc) was used for the precipitation and deposition of Mn or Fe oxides on zirconia fibers. Two types of fibers were used: commercial (Fib Zr(C)) and biomorphic (Fib Zr(B)) ZrO_2_ fibers, the latter produced using cotton as a biotemplate. Both series of supported catalysts were characterized physicochemically and morphologically. Scanning Electron Microscopy (SEM) analyses showed that Fib Zr(B) largely retained the morphology of cotton. Fib Zr(B) presented the tetragonal phase (t-ZrO_2_), while Fib Zr(C) exhibited the monoclinic phase (m-ZrO_2_). Using X-ray Diffraction (XRD), the cryptomelane phase (K_x_Mn_8_O_16_) was identified only for Mn-Fib Zr(B). In the case of Fe-supported samples, the α-Fe_2_O_3_ phase appeared clearly in both biomorphic and commercial fibers. SEM and Transmission Electron Microscopy (TEM) images revealed that the precipitated iron oxides appeared to be better distributed than the manganese oxides, covering the outer surface of the fibrous supports more homogeneously. X-ray Photoelectron Spectroscopy (XPS) confirmed that Mn has an average oxidation state between 3+ and 4+, consistent with the cryptomelane phase detected by XRD. The synthesized supported systems were tested as catalysts in soot and CO oxidation, with the Mn-supported fibers proving to be more active than their Fe-containing counterparts in both reactions.

## 1. Introduction

Air pollutants released into the atmosphere represent a major environmental concern due to their adverse effects on both ecosystems and human health. Gaseous and particulate emissions originating from industrial processes, transportation, and energy production contribute to the formation of secondary pollutants and air quality deterioration. Among these contaminants, particulate matter (PM), nitrogen oxides, sulfur dioxide, Volatile Organic Compounds (VOCs), carbon monoxide, dioxins, and polycyclic aromatic hydrocarbons (PAHs) are all considered air pollutants that are harmful to humans. Exposure to these contaminants has been directly associated with respiratory and cardiovascular diseases, as well as increased mortality rates. Therefore, developing effective strategies to mitigate atmospheric emissions is of critical importance in order to protect public health and ensure environmental sustainability [1,2].

Catalysis has been the main tool for combating pollution, mainly by its application in exhaust gases after-treatments. Through the use of catalysts, harmful compounds can be transformed into products with a lower impact factor. In the case of catalytic oxidation, contaminants can be converted into CO_2_ and H_2_O, thus diminishing their impact upon release to the atmosphere. The synthesis of catalysts commonly involves the use of supports on which active phases are added. Due to its chemical and excellent thermal stabilities, high resistance for corrosion, and biocompatibility, ZrO_2_ has been widely used as a support [3,4,5].

On the other hand, the morphology of supports plays a key role in catalytic activity. Among the different support morphologies, fibrous materials are promising as supports in the area of catalysis due to their high length-to-diameter ratio, high surface-to-volume ratio, large fraction of voids, elevated mass transfer, easy scale-up, and low cost [6]. A very simple and innovative method for synthesizing fibers is the biotemplating technique, which allows us to transform, usually by thermal treatments, organic biotemplates into inorganic structures, the latter maintaining the unique morphologies found in nature. Additionally, discarded fibers can also be used as structural generators, thus enabling the recycling of materials.

Regarding catalytic materials, manganese and iron oxides are considered good catalysts for oxidation reactions due to their redox capabilities, variety of oxidation states, abundance, low cost, and good stability under oxidizing conditions. In the case of manganese oxides, their ability to alternate between the Mn^2+^, Mn^3+^, Mn^4+^, and Mn^≥4+^ states allows them to donate and recapture oxygen from the crystal lattice, favoring Mars van Krevelen-type mechanisms, as demonstrated in the oxidation of CO on nanocrystalline MnOx with high surface area [7]. In addition, different structures (such as layered or large-tunnel structures) show significantly better oxidation performances than those of compact 3D networks due to the morphology and accessibility to bulk oxygen [8].

On the other hand, iron oxides (FeOx) also exhibit useful redox properties, especially due to the coexistence of Fe^2+^ and Fe^3+^. The oxidation of carbon monoxide on pure α-Fe_2_O_3_ has been studied, and the kinetics showed that oxygen adsorbed on the oxide surface plays a key role and that the oxygen binding energy is a determining parameter for activity [9]. Also, Fe_2_O_3_/Fe_3_O_4_ catalysts have been studied for carbon particle oxidation. According to Weinland et al. [10], the use of Fe_3_O_4_ nanoparticles resulted in a reduction in activation energy, and consequently, low carbon oxidation temperatures were obtained for different types of carbon nanoparticles. Moreover, the addition of Fe_2_O_3_ to CeO_2_ resulted in active and thermally stable catalysts for soot oxidation [11].

There are several techniques used for depositing catalysts on a suitable support, where a good dispersion of the active phase is desired in order to promote the contact of the catalyst with the contaminant and, therefore, the catalytic reaction. In this sense, the gliding arc plasma technique (glidarc) offers an interesting and innovative approach to deposit active phases on fibers as the precipitation of metallic oxides can be favored [12]. Plasma can be generated when humid air is passed through a nozzle and ionized by applying a high voltage between two electrodes. In this way, very reactive species like radicals and electrons are formed, which allow specific reactions in the liquid phase to take place and can enhance conventional chemical processes. As a matter of fact, classical precipitation methods require co-reactants (acids or bases) with the subsequent controlled addition, whereas one of the advantages of this novel technique is that precipitation agents are not needed. However, the glidarc technique is limited to the precipitation of thermodynamically favored oxides.

In this context, the objective of this work is to study the plasma-assisted precipitation method (glidarc technique) to develop supported catalytic films using inorganic fibers as supports. The latter are ZrO_2_ fibers either prepared by the biotemplate route or commercial ones. The catalytic phases, based on Fe or Mn oxides, are added by glidarc on ZrO_2_ fibers. The supported catalysts were used for soot and CO removal, which constitute two dangerous pollutants present in the atmosphere that come from mobile and stationary sources due to incomplete oxidation reactions. Several characterization techniques are used to study the properties of the developed catalysts and their effect on catalytic performance.

## 2. Materials and Methods

### 2.1. Synthesis of Biomorphic Fibers

The method used for the obtention of biomorphic fibers was biotemplating [13,14]. This consisted of the incipient wet impregnation (IWI) of a biological template (commercial cotton) with a solution of the precursor salt. For this purpose, 2.5 g of cotton was put in contact with a solution of ZrO(NO_3_)_2_ (N_2_O_7_Zr.xH_2_O Aldrich Chemistry, St. Louis, MO, USA) in an amount that ensured the total absorption of all the liquid (30 mL). The impregnated cotton was then dried at 100 °C for 1.5 h and calcined at 600 °C (10 °C/min) in air for 5 h. The concentration of the ZrO(NO_3_)_2_ solution was calculated to obtain 1 g of fibers, after calcination. The zirconia fibers obtained by this route were denominated Fib Zr(B), whereas commercial fibers (Zircar, Florida, NY, USA, zirconia 99%) were named Fib Zr(C) and used for comparison purposes.

### 2.2. Preparation of Catalytic Fibers by Glidarc Plasma

**Figure 1:**
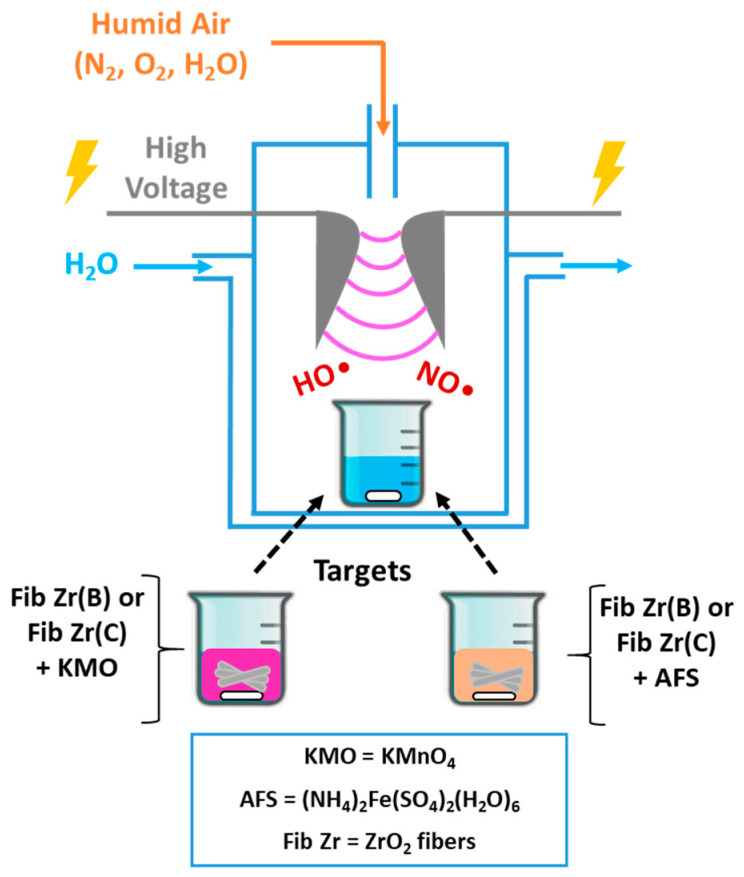
A scheme of the glidarc plasma set-up used to deposit Fe or Mn oxides over commercial (C) or biomorphic (B) ZrO_2_ fibers.

Figure 1 depicts the glidarc plasma homemade set-up used along with the different possible targets exposed [12,13].

*Plasma generation and target exposure:* The core of the system is a glass reactor with two closely spaced (2–3 mm), rounded, diverging electrodes. These electrodes are connected to a high-voltage transformer (220 V/9 kV). When activated, the transformer generates an arc at the point of the smallest electrode separation. Humid air is used as the fed gas, produced by bubbling compressed air through water. A rapid increase in the gas flow rate from 1 to 5–6 L/min causes the arc to be pushed along the electrodes, break, and reform. Both the high voltage selected and the humid air flow rate are key parameters for this technique. This continuous process creates a “plume” of arcs, which constitutes the plasma and is maintained throughout the experiment. Targets are placed inside a 250 mL beaker, positioned 2–3 cm away from the plasma plume.

*Target materials:* The targets are prepared by placing 250 mg of ZrO_2_ fibers in the beaker and adding precursor solutions (100 mL) corresponding to the active phases to be deposited: KMnO_4_ solution (KMO) 0.75 g/L, for manganese oxides, or Mohr’s salt ((NH_4_)_2_Fe(SO_4_)_2_(H_2_O)_6_) solution (AFS) 5 g/L, for iron oxides.

*Post-exposure processing:* After exposure to the plasma (30 min for Mn targets or 60 min for Fe targets, times determined experimentally for precursor precipitation [13]), the solid phase is separated. The mixture undergoes centrifugation for 1 h at 14,000 rpm (equivalent to 22,830 g RCF) using a HERAEUS Multifuge X1R centrifuge (Thermo Fisher Scientific, Waltham, MA, USA), and then the separated solid is dried 2 h at 120 °C and calcined at 600 °C for 2 h in air flow. It is convenient to clarify that not all the precursor contained in the beaker is precipitated and also that not all amounts of oxides precipitate over ceramic fibers.

### 2.3. Characterization Techniques

#### 2.3.1. X-Ray Diffraction (XRD)

Crystalline phases were detected using a Bruker-D8 Advance diffractometer (Billerica, MA, USA) with Bragg–Brentano geometry and a LynxEye XE-T detector with Cu Kα radiation (λ = 0.15418 nm, 40 kV, 30 mA). Samples were scanned from 2θ = 5° to 80° at a scan rate of 2.2°/min. Phase identification was conducted using Panalytical Highscore software (Version 4.8). The Scherrer equation was used to calculate crystallite sizes.

#### 2.3.2. N_2_ Physisorption

Micromeritics Tristar 3000 equipment (Norcross, GA, USA) was used, degassing samples under vacuum (6.6 Pa) at 170 °C for 3–4 h prior to the analyses. The measurements were carried out at −196 °C at a relative pressure (p/p_0_) range of 0.01–0.99. The specific surface area values (S_BET_) were calculated by means of the Brunauer–Emmet–Teller (BET) method.

#### 2.3.3. Scanning Electron Microscopy (SEM) and Energy-Dispersive X-Ray (EDS) Analyses

SEM images we acquired using Carl Zeiss Sigma SEM equipment with EDS (OXFORD-AZTEX XMAX 80, Jena, Germany) operating at a voltage of 20 kV. The samples were covered with a gold film (8 nm thick). The EDS analysis depth was 1 μm.

#### 2.3.4. Transmission Electron Microscopy (TEM)

A JEOL JEM-2100Plus microscope (Tokyo, Japan) was used to acquire TEM images at an accelerating voltage of 200 kV. Samples were dispersed in isopropyl alcohol, and then drops were added onto carbon-coated copper grids (300 mesh). To calculate particle sizes from the TEM images, the open-source software Gatan Digital Micrograph version 2.32.888.0 was used.

#### 2.3.5. Inductively Coupled Plasma Atomic Emission Spectroscopy (ICP-AES)

The elemental compositions of the catalysts were obtained from Inductively Coupled Plasma Emission Spectrometry (ICP), using Perkin Elmer Optima 2100 equipment (sequential, Waltham, MA, USA). Prior to the analyses, samples were completely dissolved by acid digestion using a HNO_3_-HClO_4_ mixture (1–5 *v*/*v* ratio). The quantitative determinations of Fe and Mn contents were carried out using calibration curves from certified standard solutions. The emission lines selected were λ = 238.20 nm for Fe and λ = 259.37 nm for Mn.

#### 2.3.6. Laser Raman Spectroscopy (LRS)

A Horiba JOBIN YVON LabRAM HR spectrometer (Kyoto, Japan) was used to obtain sample spectra. The apparatus was equipped with a CCD detector cooled to −70 °C using the Peltier effect and coupled to an Olympus confocal microscope with a 100× objective lens. The laser power was 5 mW, and a collection time of 10 s with 10 accumulations (resolution = 4 cm^−1^) was used. The excitation wavelength was 532 nm (diode-pumped solid-state laser).

#### 2.3.7. Attenuated Total Reflection Mode (FTIR-ATR)

A Bruker FTIR Equinox 55 spectrometer with a Bruker Platinum ATR module (diamond crystal) was utilized, collecting 100 scans from both background and sample measurements, from 4000 to 400 cm^−1^ with a resolution of 4 cm^−1^, in absorbance mode. OPUS 6.5 software was used to collect data, applying an ATR extended correction to adjust the effect of lower peak intensity at longer wavenumbers and any possible shift when compared to a transmission spectrum. The correction parameters selected were the following: number of ATR reflections = 1; ATR incidence angle = 45°; and average refractive index of the sample = 1.5.

#### 2.3.8. X-Ray Photoelectron Spectroscopy (XPS)

The spectra were obtained using a multi-technique Specs equipped with a dual Ag/Al monochromatic X-ray source, model XR50, and a hemispheric analyzer PHOIBOS 150 (Berlin, Germany) in fixed analyzer transmission mode (FAT). The spectrometer was operated with a pass energy of 30 eV using the Al monochromatic anode operated at 300 W. The pressure during the measurement was lower than 10^−9^ mbar. Samples were pressed and vacuumed at 10^−3^ mbar for 10 min at 200 °C and then at ultra-high vacuum for 2 h. Conductive sample holders Specs SH 2/12 were used. A flood gun with 1 eV energy and 0.2 mA current was used to diminish surface charge effects. CasaXPS software version 2.3.23PR1.0 was used to process collected spectra. The C 1s signal (contamination carbon) at 284.6 eV was used as a reference. All spectra were processed using a Gaussian–Lorentzian (GL30) line shape and Shirley baseline.

### 2.4. Temperature Programmed Oxidation (TPO) Tests: Soot and CO Oxidation

The activity of the synthesized catalysts for soot and CO oxidation reactions was studied by Temperature Programmed Oxidation experiments.

#### 2.4.1. Soot Oxidation Tests

A CATLAB Microreactor module from HIDEN ISOCHEMA (Warrington, UK) was used. The module is coupled with a mass spectrometer (MS) for the identification and quantification of gaseous species (reactants and products). A quartz reactor was used, in which a mixture of catalyst–soot was placed, sandwiched with quartz wool. The temperature was raised from room temperature up to 80 °C, then maintained at this value for 1 h, and finally ramped up to 625 °C at a rate of 10 °C/min. The total feed flow was 30 mL/min, with a composition of 10 mL Ar/min, 1 mL O_2_/min, and 19 mL He /min. The loading of catalysts and soot mixtures (20–25 mg) was performed in order to obtain a 1 cm high catalytic bed.

Commercial carbon black Printex U was used as model soot at a fixed catalyst–soot ratio of 20-1. Catalysts were put in contact with a soot–hexane suspension under gentle stirring, maintained thanks to an oil bath at 60 °C until solvent evaporation, and finally dried at 80 °C for 2 h. This approach is called *“wet contact”* and preserves fiber morphology.

From MS signals and the gas flow used (30 mL/min), CO_2_ molar flows ($f_{{CO}_{2}}$) were calculated at different times (corresponding to different temperatures, according to the heating rate used).

The carbon conversion percentage ($x_{C(t)}$) is defined in Equation (1), where the initial time (“0”) was considered as the time at which the CO_2_ detected comes from soot burning, which depends on each sample. The final time ($t_{f}$) corresponds to the time at which the CO_2_ signal is negligible, $n_{C(t)}$ indicates the molar amount of carbon burned at time t, and $n_{C}^{T}$ indicates the total molar amount of carbon burned during the whole TPO experiment.

(1) $$x_{C(t)}=\frac{\;n_{C(t)}}{n_{C}^{T}}\cdot \;100=\frac{\int_{0}^{t}f_{{CO}_{2}}.dt}{\int_{0}^{tf}f_{{CO}_{2}}.\;dt\;}\;\cdot \;100$$

The values of T_50_ and T_90_ correspond to the temperatures at which 50 and 90% soot conversion is achieved, respectively.

The reaction rate is defined according to Equation (2), where *n*_c_′_(*t*)_ is the molar amount of carbon burned at a certain time *t* (*n_c_*_(*t*)_), divided by the weight of the catalyst used during the catalytic test. Assuming that all carbon is completely transformed into CO_2_, *n*_c_′_(*t*)_ = *n*′*_CO_*_2(*t*)_, the latter being the molar amount of CO_2_ formed at time *t*, divided by the weight of the catalyst. From CO_2_ flow profiles against temperature, the value of temperature corresponding to the maximum combustion rate (T_M_) was obtained for each catalyst, which corresponds to the temperature at which ${f^{\prime }}_{{CO}_{2}}$ is the maximum.

(2) $$r\left[\frac{mol}{mg\;cat\;.\;s}\right]=\frac{-d{n^{\prime }}_{C\left(t\right)}}{dt}=\frac{d{n^{\prime }}_{CO2\left(t\right)}}{dt}={f^{\prime }}_{{CO}_{2}}\;$$

#### 2.4.2. CO Oxidation Tests

Measurements were performed in flow equipment using a quartz reactor coupled with a gas chromatograph from Shimadzu, model GC 2014, and a Porapak Q column, using He as a carrier. The temperature ramp applied by the furnace to the reactor was 5 °C/min from room temperature to a final temperature of 600 °C. The total flow fed was 30 mL/min and was composed of 1% CO, 2% O_2_, and He (balance). The conversion profiles were obtained using the CO_2_ areas (${CO}_{2}(t)$) from the GC. The CO conversion percentage ($x_{CO(t)}$) is calculated by dividing the CO_2_ area value obtained from the chromatogram at a specific temperature during the experiment (${CO}_{2\;}(t)$) by CO° (initial CO concentration), as expressed in Equation (3).

(3) $$x_{CO(t)}=\frac{{CO}_{2}(t)}{{CO}^{{}^{\circ}}}\cdot 100$$

## 3. Results and Discussion

### 3.1. Structural Characterization

#### 3.1.1. X-Ray Diffraction and LRS Analyses

**Figure 2:**
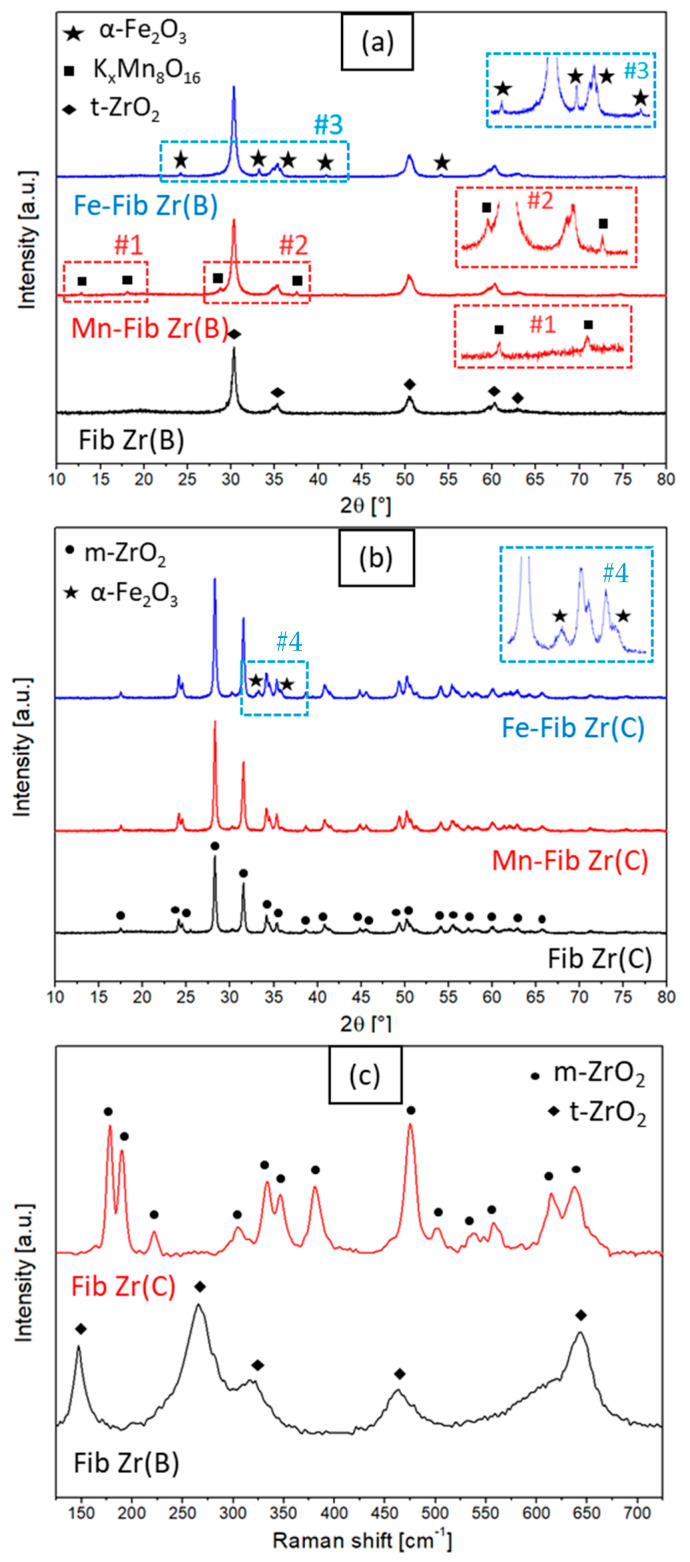
X-ray diffractograms of (**a**) Mn or Fe supported on Fib Zr(B) and bare Fib Zr(B) and (**b**) Mn or Fe supported on Fib Zr(C) and bare Fib Zr(C), and (**c**) Raman spectra of biomorphic and commercial ZrO_2_ fibers.

The diffractograms of the catalysts prepared with Mn- or Fe-supported Fib Zr(B) and Fib Zr(C) by glidarc are presented in Figure 2a and Figure 2b, respectively. It should be noted that both bare fibers show different diffractograms. Fib Zr(B) shows signals at 2θ = 30.3, 35.3, 50.4, 60.2, and 62.9° that correspond to the tetragonal phase of ZrO_2_ (t-ZrO_2_, JCPDS 17-0923), while Fib Zr(C) shows diffraction peaks corresponding to the monoclinic phase of ZrO_2_ (m-ZrO_2_, JCPDS 37-1484), with main signals at 2θ = 28.3 and 31.4°. From the Scherrer equation, the crystallite size of Fib Zr(C) calculated was 31 nm, being bigger than that of the biomorphic formulation (Fib Zr(B)), which was 19 nm.

Figure 2c shows the LRS spectra used to corroborate the phase assignations for Fib Zr(B) and Fib Zr(C) performed from XRD patterns. The Raman bands at 147, 265, 319, 463, and 642 cm^−1^ seen for Fib Zr(B) correspond to t-ZrO_2_. The Fib Zr(C) sample shows several peaks centered at 178, 190, 221, 304, 333, 346, 381, 475, 501, 536, 557, 614, and 638 cm^−1^ which correspond to m-ZrO_2_ [15,16,17,18]. It can be confirmed that the biomorphic fibers are constituted of tetragonal ZrO_2_ (t-ZrO_2_), whereas monoclinic ZrO_2_ (m-ZrO_2_) is the crystalline phase present in commercial fibers, in agreement with the XRD results.

As regards the Fe-supported samples (Figure 2a,b), either for Fib Zr(B) or Fib Zr(C) supports, many new signals appeared after the deposition of iron oxides at 2θ = 24.2, 33.2, 35.6, 40.9, and 54.1°, which match with the rhombohedral hematite phase α-Fe_2_O_3_ (JCPDS 33-0664) [19,20]. Crystallite sizes, calculated by the Scherrer equation using the peak 2θ = 33.2° for α-Fe_2_O_3_ from the diffractograms presented in Figure 2, were 21 nm for Fe-Fib Zr(C) and 35 nm for Fe-Fib(B).

On the other hand, Mn-Fib Zr(B) shows several small peaks at 2θ = 12.6, 18.3, 28.9, and 37.4°, all of which coincide with the signals found in the cryptomelane phase K_x_Mn_8_O_16_ (JCPDS 44-1386) [21,22]. Cryptomelane is part of the so-called octahedral molecular sieves (OMSs), which have tunnel structures of octahedrons [MnO_6_] that share edges. It is a mixed oxide that combines Mn^4+^ and Mn^3+^ with alkali and/or alkali earth cations that reside inside the tunnels to stabilize the structure (in this case K^+^) [22,23]. In contrast, when ZrO_2_ (C) fibers are used as a support (Figure 2b), the diffractogram for Mn-supported Fib Zr(C) shows no evidence of signals coming from MnO_x_ species, which could indicate the good dispersion of the supported phase. Moreover, the LRS spectra of the supported formulations (for both series of catalysts) were very similar to those of bare supports.

#### 3.1.2. FTIR-ATR Analyses

**Figure 3:**
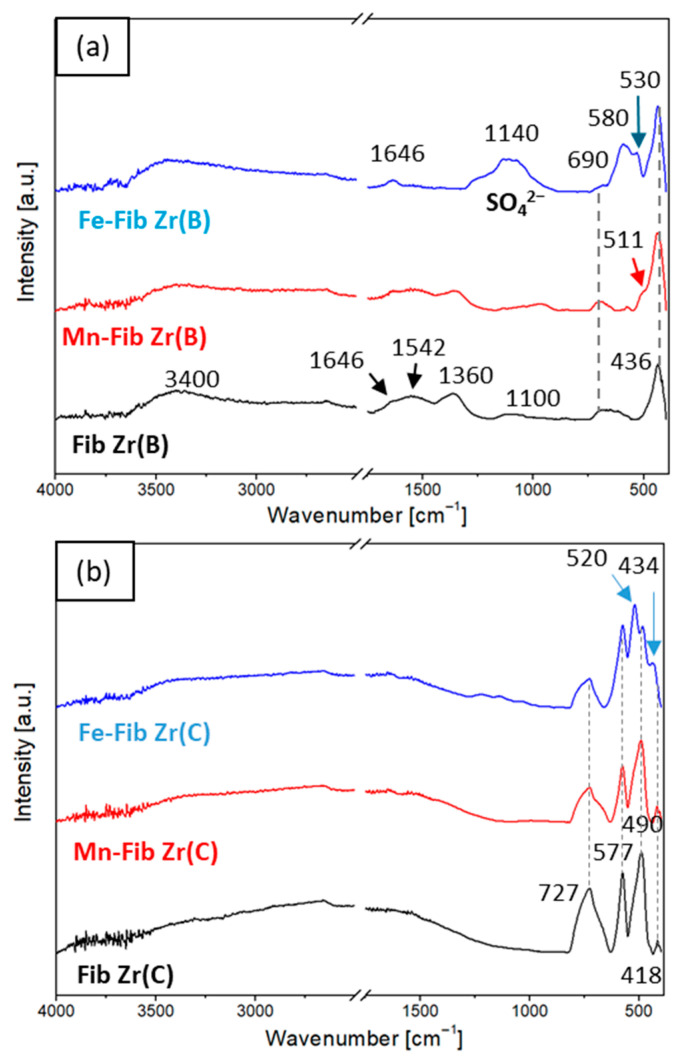
FTIR-ATR spectra of (**a**) Mn or Fe supported on Fib Zr(B) and bare Fib Zr(B) and (**b**) Mn or Fe supported on Fib Zr(C) and bare Fib Zr(C).

Figure 3a and Figure 3b shows the FTIR-ATR spectra for both series of catalysts, Fib Zr(B) and Fib Zr(C), respectively. All signal assignations made are summarized in Table 1.

All samples of Fib Zr(B) series (Figure 3a) show characteristic signals coming from water physically adsorbed on the sample: a broad band at 3350–3400 cm^−1^ due to the H-bonded -OH stretching vibration [24] and a small signal at 1646 cm^−1^ corresponding to H-O-H vibrations. The bands located at 1542, 1360, and 1100 cm^−1^ are associated with strongly adsorbed carbonates, which could originate from CO_2_ chemisorption after the template decomposition or from ambient air contact [25,26,27]. These carbonate bands are less pronounced after Mn deposition and are not present in the Fe-supported sample. In the latter, a broad band with a maximum at 1140 cm^−1^ is observed, which is related to sulfate species coming from the precursor (Mohr’s salt, AFS) and remaining in the catalyst after calcination. The absence of carbonates could be due to the displacement of these species by the sulfates [13,28,29].

In the metal–oxygen region, a broad band situated at approximately 690 cm^−1^ is present in Fib Zr(B) and also for both supported samples. As reported and according to the XRD and LRS results, this band is assigned to Zr-O vibrations from t-ZrO_2_ [30]. In addition, another band at 436 cm^−1^ is observed both for bare and catalytic biomorphic samples, also coming from Zr-O vibrations of t-ZrO_2_ [31].

For Mn-Fib Zr(B), a clear shoulder appears at 511 cm^−1^ that matches with Mn-O vibrations in the cryptomelane structure. Other peaks at 462 and 682 cm^−1^ are expected from the stretching modes of the MnO_6_ octahedron of cryptomelane, which are probably overlapped by t-ZrO_2_ signals [22].

As regards Fe-Fib Zr(B), two new bands can be seen at 580 and 530 cm^−1^, which correspond to Fe-O stretching vibrations, the latter ascribed to the α-Fe_2_O_3_ phase and the former to Fe_3_O_4_ [19,32]. The other signal expected for the α-Fe_2_O_3_ phase should appear around 434 cm^−1^, but in this case, it is probably overlapped with the intense Zr-O vibration peak. Although the magnetite phase was not detected by XRD, it should be considered that the FTIR-ATR technique is more superficial.

For the series of commercial fibers (Figure 3b), no signals due to either physiosorbed water or carbonates are seen. Fib Zr(C) presents three strong absorption bands at 727, 577, and 490 cm^−1^, along with a weak peak at 418 cm^−1^, which are all attributed to Zr-O stretching vibrations in the m-ZrO_2_ structure [33].

No signal could be assigned to MnO_x_ species in Mn-Fib Zr(C), which could be masked by the intense Zr-O bands. Regarding Fe-Fib Zr(C), two new signals (if comparing to the spectrum of the bare support) are evident at 520 and 434 cm^−1^, which are typical of the α-Fe_2_O_3_ phase [20,34]. The peak around 580 cm^−1^ from Fe_3_O_4_ (if present), seen for Fe-Fib Zr(B), could be masked in the spectrum of Fe-Fib Zr(C) by the ZrO_2_ peak at 577 cm^−1^. Also, if comparing Figure 3a,b, it can be noticed that the surface sulfate signals are weaker for Fe-Fib Zr(C).

### 3.2. Chemical, Morphological, and Textural Properties

#### 3.2.1. Scanning Electron Microscopy (SEM) and Energy-Dispersive X-Ray (EDS) Analysis

**Figure 4:**
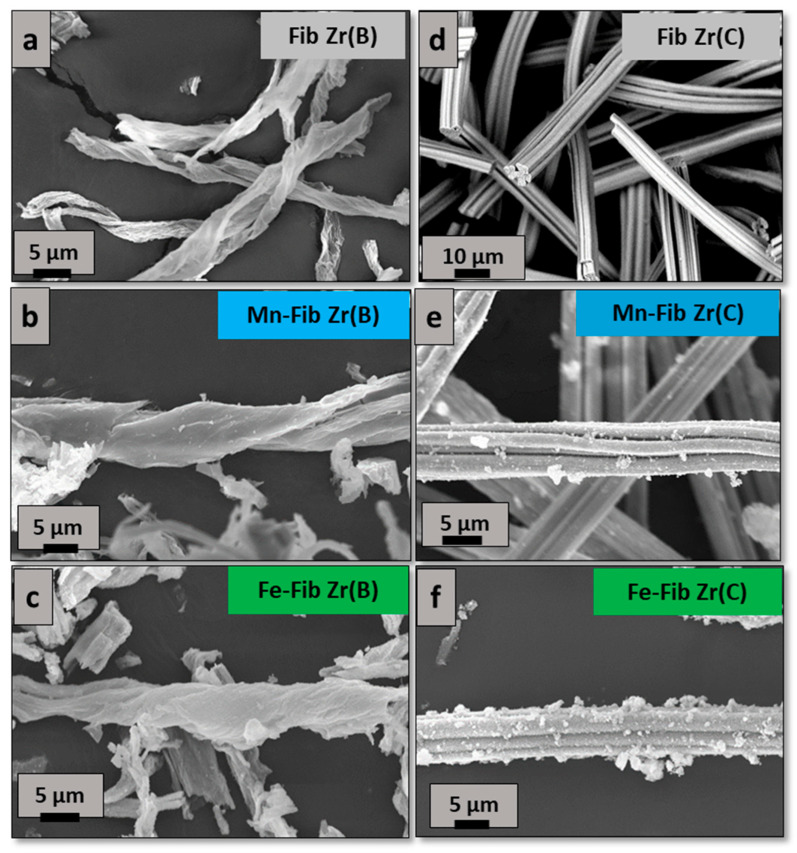
SEM images of both series synthesized of fibrous catalysts. (**a**–**c**) catalysts prepared from biomorphic fibers and (**d**–**f**) catalysts prepared using commercial fibers.

SEM images obtained from biomorphic and commercial zirconia fibers (Fib Zr(B) and Fib Zr(C)) are shown in Figure 4a and Figure 4d, respectively. For the former, the template morphology was highly maintained. Translucid fibers (1 μm thick) are formed (Figure 4a), resembling the cotton cellulosic fibers [13]. Their lengths reach up to 80 μm and their widths are about 5–10 μm. Most fibers are open-helical, and closed ones were almost not seen.

On the other hand, Fib Zr(C) appears as bundles of several small cylindrical fibers, with uniform lengths of 80–100 μm and widths of 5–10 μm approximately (Figure 4d). As can be clearly observed, these fibers are more regular in shape and size than the biomorphic ones.

In Figure 4b,e or Figure 4c,f, when Mn or Fe is added, respectively, small aggregates (<1 μm) are observed in the case of catalysts supported on Fib Zr(C), being more abundant for the iron sample (Fe-Fib Zr(C)). However, for catalysts supported on Fib Zr(B), catalytic aggregates are not clearly distinguished.

**Figure 5:**
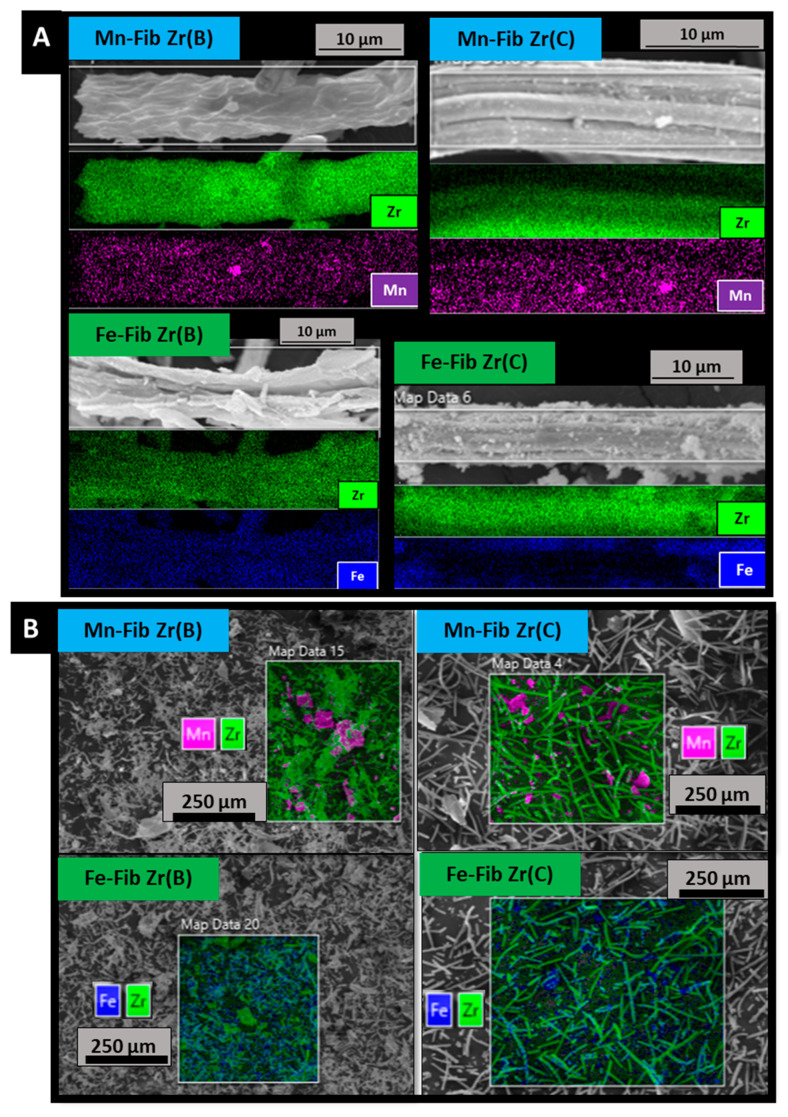
EDS fiber mapping (**A**) and big mapping (**B**) analyses of catalytic samples.

EDS analyses (Figure 5A,B) allowed us to quantify metallic contents. Two different types of quantifications were carried out for all samples: fiber mappings (Figure 5A) and big mappings (Figure 5B). All values obtained from this technique are reported as atomic metal ratios in Table 2, for both Fib Zr(B) and Fib Zr(C) series of catalysts.

Figure 5A shows that both metals distribute all along zirconia fibers. EDS was able to detect the presence of manganese or iron on the biomorphic fibers, which could not be clearly distinguished in the SEM images (Figure 4). The big mapping images (Figure 5B) show more and bigger agglomerates of Mn in Mn-Fib Zr(B) and Mn-Fib Zr(C), whereas the Fe-supported samples seem to have a better distribution of the precipitate without agglomerations.

From Table 2 it can be observed that manganese is heterogeneously deposited on zirconia fibers, either biomorphic or commercial ones, as can be inferred if fiber and big mappings are compared. On the other hand, it can be noticed that iron appears more homogeneously distributed on both types of fibers. Metallic contents, wt.%, obtained from ICP analyses (also included in Table 2), show the same trend. Both techniques show that more amounts of iron are precipitated by the plasma technique, for both biomorphic and commercial zirconia fibers.

#### 3.2.2. Transmission Electron Microscopy (TEM) Analysis

**Figure 6:**
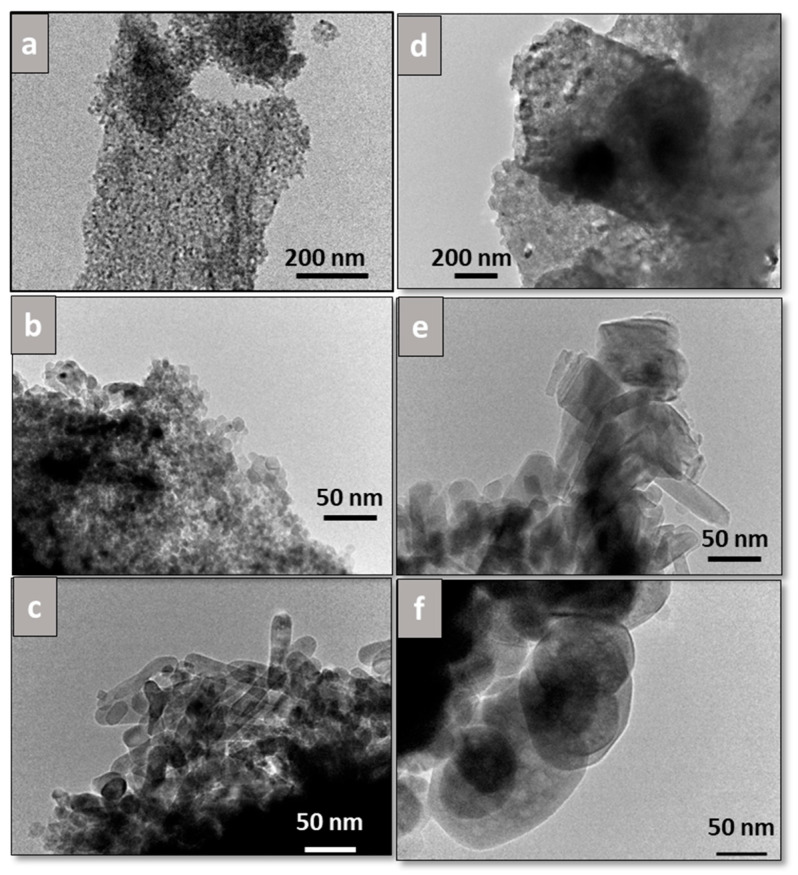
TEM images of (**a**) Fib Zr(B), (**b**) Mn-Fib Zr(B), (**c**) Fe-Fib Zr(B), (**d**) Fib Zr(C), (**e**) Mn-Fib Zr(C), and (**f**) Fe-Fib Zr(C).

TEM images for all catalysts are shown in Figure 6, from which particles were measured, and average values from at least 50 particles were obtained. Bare Fib Zr(B) (Figure 6a) exhibits rounded-edge particles 13.6 ± 3.8 nm in size, whereas Fib Zr(C) appears as bigger particles, 69.8 ± 22.9 nm in size, with marked edges (Figure 6d). For Mn-Fib Zr(B), the same morphology as that of the support is observed, along with the appearance of bigger rounded particles (Figure 6b). On the contrary, for Fe-Fib Zr(B), a different morphology can be appreciated, consisting of short rods of 19.9 ± 3.8 nm width and about 60 nm length (Figure 6c).

In the case of supported commercial fibers, Mn-Fib Zr(C) maintains the morphology of the bare fibers, also showing lengthened particles (rods), probably ascribed to manganese oxides (Figure 6e). For Fe-Fib Zr(C), it can be noticed that the morphology is similar to that of Fib Zr(C), with the presence of slightly bigger particles with more rounded edges (Figure 6f).

It is worth noticing that rods are obtained for both Fe-Fib Zr(B) and Mn-Fib Zr(C), being more rounded in the former and with more marked edges in the latter.

**Figure 7:**
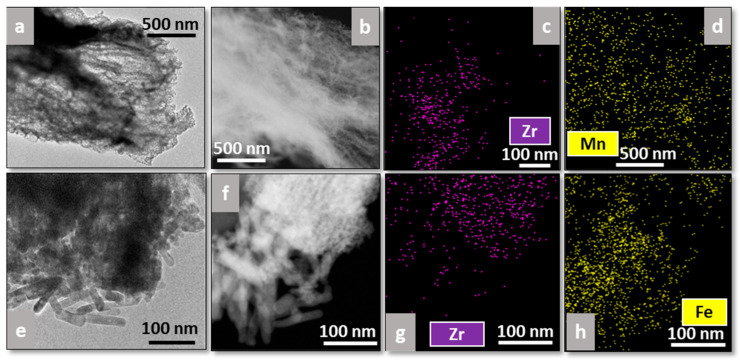
TEM images and EDS analyses of Mn-Fib Zr(B) (**a**–**d**) and Fe-Fib Zr(B) (**e**–**h**).

**Figure 8:**
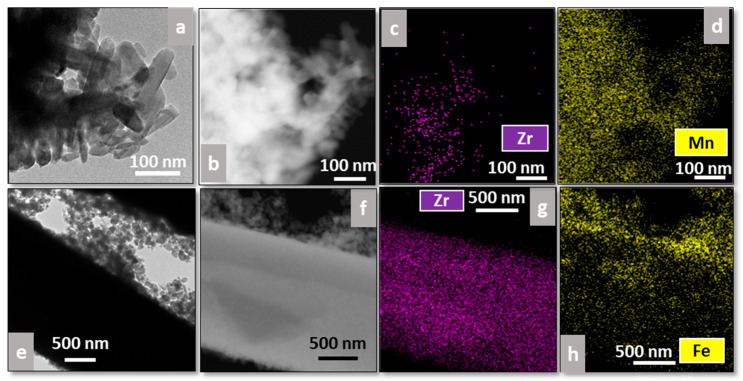
TEM images and EDS analyses of Mn-Fib Zr(C) (**a**–**d**) and Fe-Fib Zr(C) (**e**–**h**).

Additionally, EDS analysis was performed on all catalysts to study the distribution of the plasma-deposited elements (Mn or Fe) on the supports (Figure 7 and Figure 8), where Figure 7a,e and Figure 8a,e correspond to bright-field TEM images, whereas Figure 7b,f and Figure 8b,f correspond to dark-field ones.

As regards the biomorphic fibers, Mn appears well distributed all along Fib Zr(B), as can be observed in Figure 7d, whereas in the case of Fe-Fib Zr(B), Fe appears to be more concentrated on the rod zone (bottom part of Figure 7h). The rod morphology could be linked to plasma-precipitated Fe oxides.

Regarding commercial fibers, Figure 8d shows that Mn oxides are distributed throughout the sample, appearing more concentrated on the rods (Figure 8a). This agrees with what was previously observed in Figure 6e, for which marked-edge rods could be associated with Mn species. On the other hand, for Fe-Fib Zr(C), Fe completely covers the zirconia fiber (Figure 8e–h).

#### 3.2.3. N_2_ Adsorption

**Figure 9:**
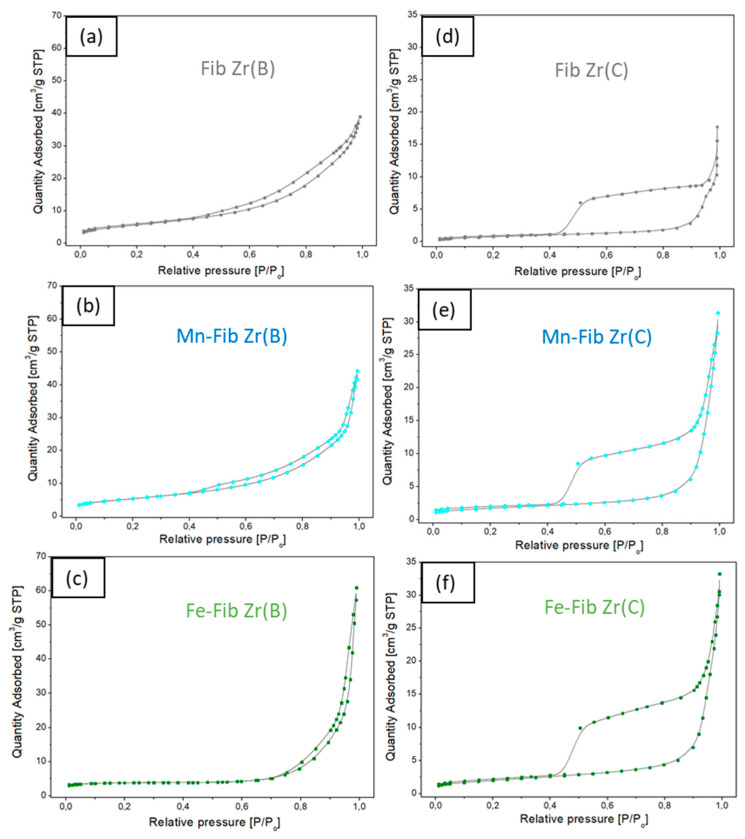
Nitrogen adsorption–desorption isotherm curves. (**a**–**c**) catalysts prepared from biomorphic fibers and (**d**–**f**) catalysts prepared using commercial fibers.

In Figure 9, the adsorption isotherms for all samples are shown. Fib Zr(B) and Mn-Fib Zr(B) (Figure 9a and Figure 9b, respectively) exhibit a similar pattern, following a type IV isotherm (which may indicate that these samples have some cylindrical mesopores opened at both ends or bottle-like mesopores), forming a hysteresis loop that ends around P/P_o_ = 0.4, being more pronounced for the former. The loop covers a wide range of pressures, which indicates that whatever their shape, pores present a marked diversity of sizes. The isotherm shows an H3-type loop, which is formed by non-uniform, plate-shaped aggregated particles forming laminar pores that often cover the macropore range [35,36].

The Fe Fib Zr(B) sample (Figure 9c), in turn, is matched with a type II isotherm (nonporous or macroporous adsorbents), where the small hysteresis loop occurs at high relative pressures (very similar emptying mechanism closing at P/P_o_ = 0.75), which may be provoked by interparticle mesoporosity caused by bigger particles.

On the other hand, the N_2_ adsorption isotherms of bare and supported commercial fibers exhibit a type II isotherm, all of them exhibiting a huge type H4 hysteresis loop that ends around P/P_o_ = 0.4 (Figure 9d–f). This type of loop is associated with solids consisting of aggregates or agglomerates of particles forming slit-shaped pores with uniform size and/or shape [35]. This could be expected as commercial fibers are very regular in shape, as could be observed in SEM micrographs (Figure 4d). For both biomorphic and commercial fibers, it can be inferred that they do not show any microporosity due to the lack of N_2_ adsorption at low relative pressures.

In terms of specific surface area (SSA), when Mn was added to Fib Zr(B), the SSA of this sample remained similar to that of the bare support (20 m^2^/g), whereas after Fe deposition, the value decreased to 12 m^2^/g. Meanwhile, Fib Zr(C) showed a very low SSA value of 3 m^2^/g, and the supported samples, either with Mn or Fe, slightly increased the SSA to 6–7 m^2^/g. This small increase in SSA could be originated by the porosity of the deposited oxide.

### 3.3. Surface Analyses

The C 1s, O 1s, Zr 3d, Mn 3s, Mn 2p, and Fe 2p XPS core regions were studied to gain insights into the surface characteristics of supported catalysts.

**Figure 10:**
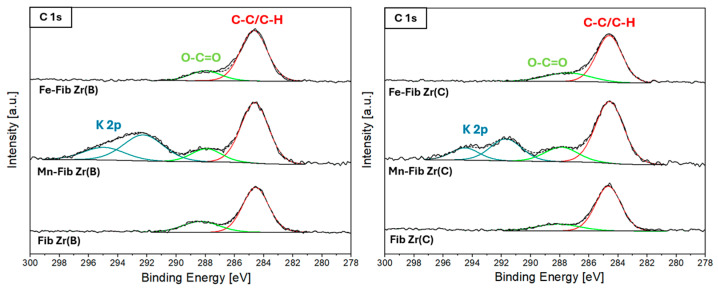
XPS C1s region for Fib Zr(B) catalysts (**left**) and Fib Zr(C) catalysts (**right**).

Figure 10 shows the C 1s region for all catalysts. The main peak due to carbon in C-(C,H) bonds at 284.6 eV (reference), along with a less intense peak around 288.6 eV (carbonate species), appears in all six samples. For the supported samples containing Mn, Mn-Fib Zr(B) and Mn-Fib Zr(C), two more signals are present at higher binding energies which correspond to a K 2p spin–orbit doublet (K 2p_3/2_-2p_1/2_), which comes from the precursor used in synthesis (KMO).

Regarding the O 1s core level (Figure S1), all samples were fitted using two peaks: a main peak between 529.3 and 529.8 eV corresponding to lattice oxygen (O_latt_) and a second peak characteristic of chemisorbed oxygen species (O_2_^−^, O_2_^2−^, O^−^, CO_3_^2−^) [37,38]. All atomic ratio values derived from these deconvolutions are listed in Table 3.

The amount of O_ads_ (and consequently the O_ads_/O_latt_ ratio) decreased after Mn addition and increased after Fe deposition for both supports, with this effect being a bit more pronounced for the biomorphic fibers. The Fe-containing samples showed a marked increase in the O_ads_/Zr ratio, with both Fe-Fib Zr(B) and Fe-Fib Zr(C) reaching similar values. It appears that the presence of α-Fe_2_O_3_ on the surface provides a considerable amount of O_ads_ species. Mn-Fib Zr(B) did not affect the original O_ads_/Zr ratio of the support much, whereas Mn-Fib Zr(C) suffered from a marked increase to a value of 1. When comparing metal ratios (M/Zr), a higher amount of Mn accumulated on the surface of Fib Zr(C) than on Fib Zr(B). This is in line with the EDS results shown in Table 2. Concerning Fe-supported samples, both of them showed considerable increments in surface M/Zr ratios with respect to the Mn-supported catalyst, evidencing that there is more Fe than Zr on the surface of both catalysts. Again, this agrees with the values presented in Table 2.

The binding energy values of Zr 3d_5/2_ and Zr 3d_3/2_ are summarized in Table 3. The peaks corresponding to Zr 3d_5/2_ appeared at 181.7–182.2 eV and their spin–orbit couples for Zr 3d_3/2_ at 184.1–184.6 eV, separated 2.3–2.4 eV from each other, which agree with the presence of Zr^4+^ [15,39].

**Figure 11:**
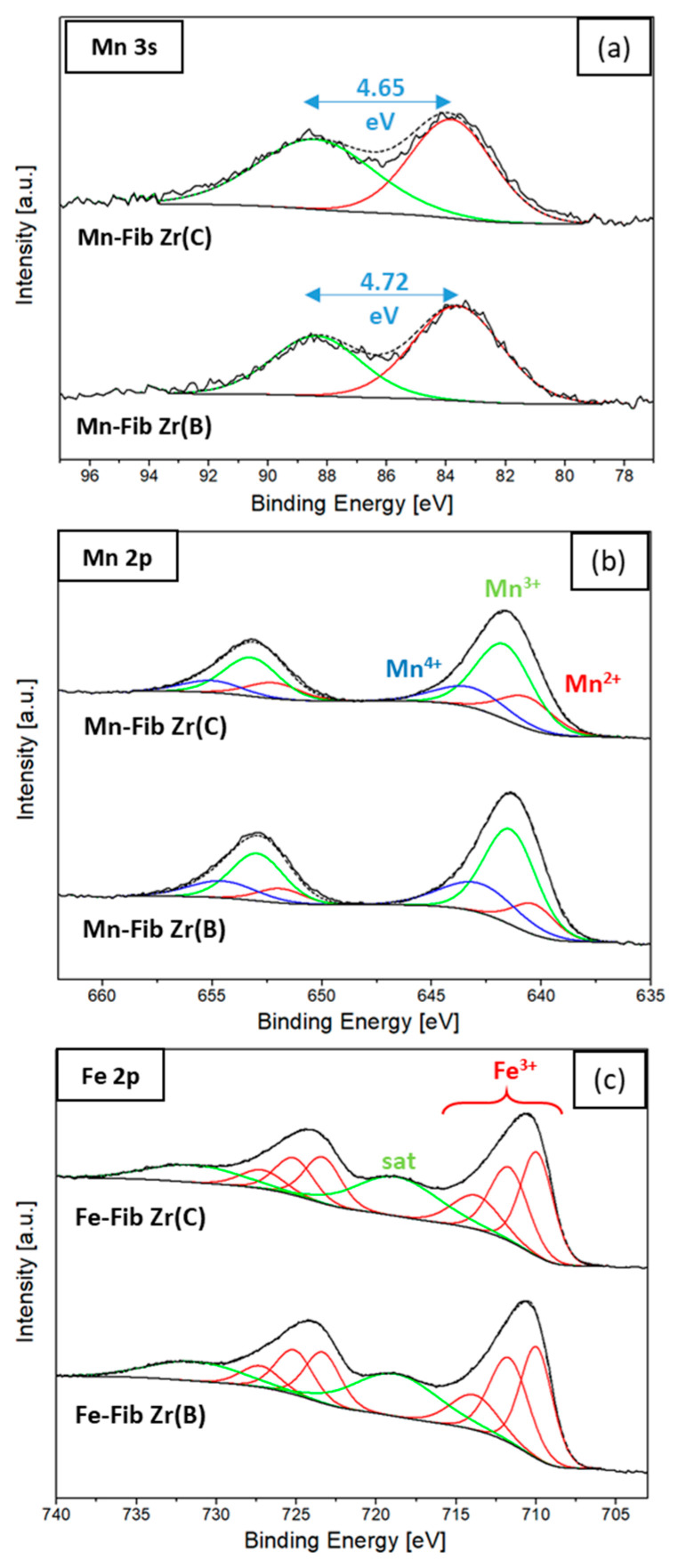
Surface species of Mn and Fe (XPS): (**a**) Mn 3s, (**b**) Mn 2p, and (**c**) Fe 2p regions.

Both Mn 3s and Mn 2p signals were collected to study surface Mn species. Figure 11a shows Mn 3s signals, where the main signal and the corresponding spin–orbit splitting (ΔBE) are observed.

The peak separation (ΔBE) found in the Mn 3s region was used in applying Equation (4) to calculate the average oxidation state (AOS) of Mn [40,41].

(4) AOS=9.67−1.27×∆BE

The AOS values obtained were 3.70 and 3.75 for Mn-Fib Zr(B) and Mn-Fib Zr(C), respectively. These results prove the coexistence of Mn^3+^ and Mn^4+^ species in these catalysts.

The fitting of Mn 2p regions for both Mn-containing samples is shown in Figure 11b. The main peaks of Mn 2p_3/2_-2p_1/2_ presented a binding energy gap (ΔE_1_) of about 11.6 eV. After peak fitting, the resulting binding energies for Mn^2+^, Mn^3+^, and Mn^4+^ species varied between 640.6 and 640.3 eV, 641.7 and 641.4 eV, and 643.2 and 642.9 eV, respectively [42,43].

The XPS spectra for the Fe-containing samples are depicted in Figure 11c. The binding energy separation between Fe 2p_3/2_ and 2p_1/2_ (ΔE_2_) was 13.5 eV, and several peaks centered at 710, 711.7, and 713.8 eV were assigned to Fe^3+^ species (multiple splitting), along with a satellite at 718.7 eV [44,45,46], in agreement with the XRD results (Figure 2).

### 3.4. Catalytic Evaluations—Soot Combustion

#### 3.4.1. Soot Combustion Tests

**Figure 12:**
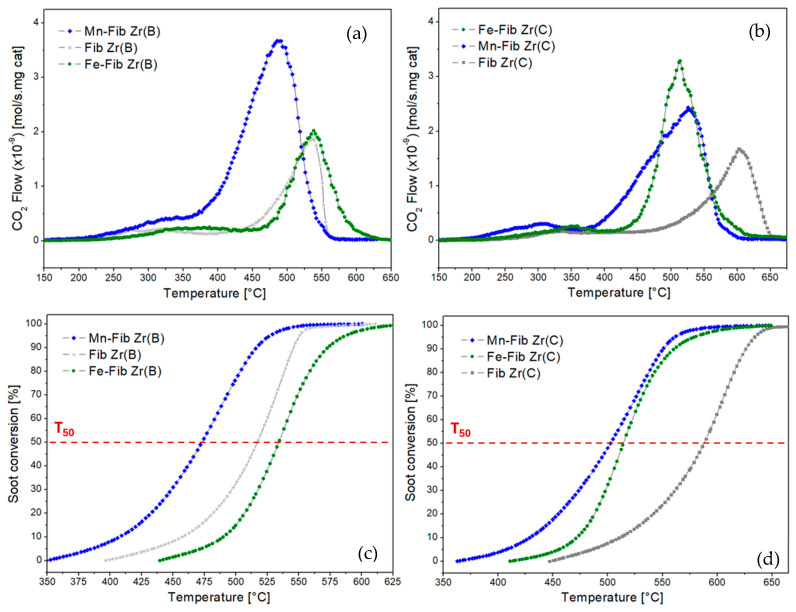
TPO and conversion profiles for Mn,Fe-Fib Zr(B) (**a**,**c**) and Mn,Fe-Fib Zr(C) (**b**,**d**) catalysts.

The reaction rate values and soot conversion profiles derived from TPO tests for all samples are presented in Figure 12, where “a” and “c” correspond to the biomorphic fiber samples, while “b” and “d” correspond to the commercial fiber samples. The corresponding T_50_, T_90_, and T_M_ values extracted from the graphs are summarized in Table 4. It is important to remark that catalytic tests were carried out in the absence of NO in the feed (only using diluted oxygen) for which the activities of the studied catalysts are expected to be better in the presence of NO, which is the common case of diesel engine exhausts.

Among the biomorphic series of catalysts, the best-performing sample was Mn-Fib Zr(B) with a T_M_ of 493 °C. Fe-Fib Zr(B) and Fib Zr(B) showed similar T_M_ values, but the latter gave better T_50_ and T_90_ values. The addition of Fe to this support practically did not have any positive effect on activity.

Regarding the series of commercial fibers, both Mn- and Fe-supported samples produced a considerably lower T_M_ value of the support in a similar manner (from 600 °C to around 500–520 °C). This could probably be ascribed to the slight increase in the specific surface area of both Mn-Fib (C) and Fe-Fib(C) (6–7 m^2^/g versus 3 m^2^/g for the bare commercial fibers), as higher SSA values benefit soot-to-catalyst contact, which is a key factor in soot combustion. However, it should be considered that the catalytic activity of bare Fib Zr(C) is considerably lower than that of bare biomorphic ZrO_2_ fibers and that the addition of either Mn or Fe makes TPO profiles shift to lower temperatures, with CO_2_ profiles being comparable to those exhibited for Fe-Fib Zr(B) and Fib Zr(B).

As TEM and SEM studies have shown (Figure 4 and Figure 6), biomorphic fibers are constituted by smaller particles and exhibit meso- and macroporosity that probably enhances the contact between soot and the fibrous supports, thus benefiting soot oxidation. Also, the morphology of biomorphic fibers probably helps in the contact between soot and the catalytic species. Additionally, biomorphic fibers exhibited the tetragonal phase along with a smaller crystallite size (19 nm) if compared to commercial fibers, constituted by bigger particles (31 nm) of monoclinic ZrO_2_.

It is important to consider that Mn-supported fibers contain K, as detected by XPS. As reported, alkali metals can enhance the ability of catalysts to release active oxygen species, and they can also form low-melting-point compounds, which can wet the soot surface, increasing soot-to-catalyst contact [47,48,49]. However, in this type of catalyst, the K retained inside cryptomelane tunnels will not be completely accessible to soot particles. Nevertheless, as Table 4 shows, T_M_ for Mn-Fib Zr(B) is lower than T_M_ for Mn-Fib Zr(C) (493 °C and 525 °C, respectively), which could probably be linked to the morphology and higher specific surface area of the former.

On the other hand, if Fe-containing fibers are compared, Fe-Fib Zr(C) was shown to be more active towards soot combustion than Fe-Fib Zr(B), which could be ascribed to the smaller crystallite size of the former. Contrary to what was observed for the biomorphic catalyst, an improvement in T_M_ was observed, which could probably be related to the smaller crystallite size of the hematite particles (21 nm) of Fe-Fib Zr(C) if compared to that of the α-Fe_2_O_3_ particles (35 nm) of Fe-Fib Zr(B).

Table 5 shows a comparison of the activity of the best catalysts with others corresponding to the formulations reported in the literature. This comparison shows that the catalysts reported here exhibit good activity. However, as indicated in Table 5, these values are strongly influenced by several factors, including the composition of the reactor feed, the type of soot used, and the soot-to-catalyst ratio. All these factors make comparison difficult.

#### 3.4.2. Catalytic Evaluations—CO Oxidation

The oxidation of CO is considered to proceed according to the Mars van Krevelen (MVK) mechanism, where the reaction occurs between the surface lattice oxygen of the catalyst and CO molecules. This step results in the generation of oxygen vacancies which are then filled in either with oxygen from the gas phase or by bulk oxygen.

**Figure 13:**
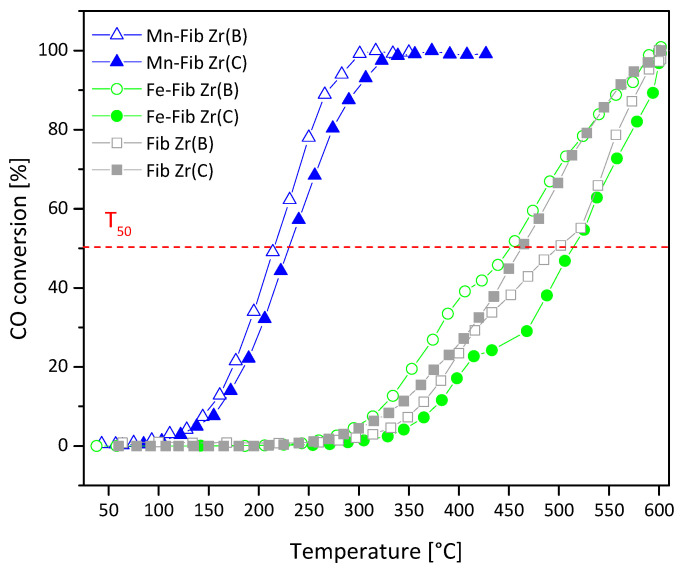
CO conversion profiles for Mn,Fe-Fib Zr(B) (empty symbols) and Mn,Fe-Fib Zr(C) (filled symbols) catalysts.

The conversion versus temperature profiles of CO oxidation experiments for all samples are presented in Figure 13, and the corresponding T_50_ and T_100_ values are listed in Table 4.

Both Fib Zr(B) and Fib Zr(C) perform badly in this reaction, with their T_50_ values being 500 °C and 464 °C, respectively. In spite of the different bulk compositions (crystalline phases), the surface composition of these two samples is similar (Table 3). Their bad catalytic performance could be linked to the poor redox capacity of ZrO_2_.

The addition of iron to either Fib Zr(B) or Fib Zr(C) did not improve catalytic activity. Although hematite is considered to be able to reversibly exchange lattice oxygen and reoxidize in air through the redox couple Fe^3+^/Fe^2+^, when supported on either Fib Zr(B) or Fib Zr(C), no positive catalytic effect could be observed. The sulfate residues detected in the iron catalysts, originating from the decomposition of Mohr’s salt, are likely playing a negative role here.

On the other hand, the Mn-containing catalysts (Mn-Fib Zr(B) or Mn-Fib Zr(C)) significantly reduce T_50_ values. The high activity of these Mn-containing catalysts could be related to the presence of the cryptomelane structure, detected by XRD and XPS. Contrary to the case of soot oxidation, the gaseous CO reactant can get inside the cryptomelane tunnels, which could help CO oxidation via a greater interaction between the reactant and oxygen active species.

In spite of the better distribution of iron oxide particles on both types of ZrO_2_ fibers (biomorphic and commercial), the activity of Fe samples is lower, which is probably linked to the poor intrinsic activity of Fe_2_O_3_.

Table 6 shows a comparison of the activities (T_50_) of the best-performing catalysts (i.e., those containing Mn) with other oxide-type catalysts reported in the literature. As observed, the activity of the Mn-ZrO_2_ fibers reported here is comparable to that of the other oxides. As Table 3 shows, these samples exhibited a higher percentage of lattice oxygen (lower O_ads_/O_latt_ ratio). Probably, the ability to alternate between Mn^2+^, Mn^3+^, and Mn^4+^ species allows for the release and recapture of oxygen from the crystal lattice, favoring CO oxidation (Mars van Krevelen mechanism). This oxygen exchange would also be favored by the accessibility to bulk oxygen of OMS tunnel structures.

#### 3.4.3. CO Oxidation: Stability Tests

**Figure 14:**
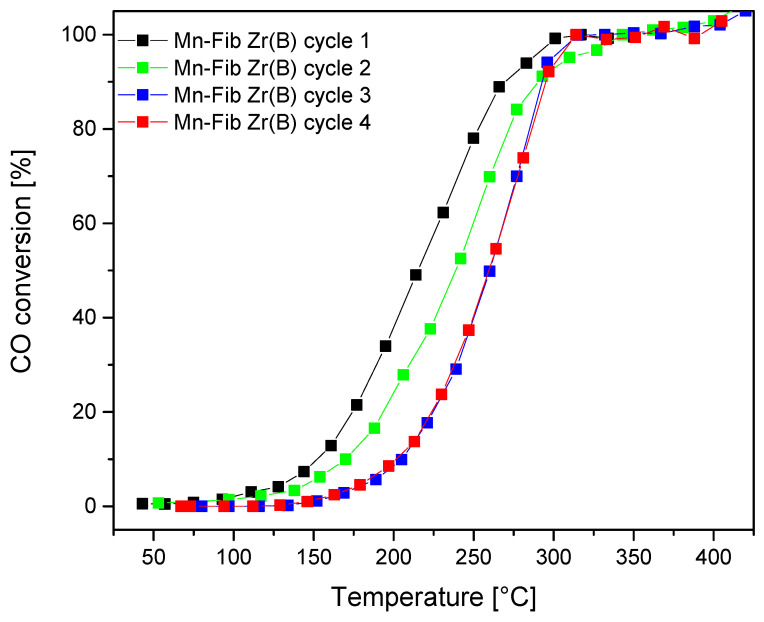
CO oxidation: Stability runs for Mn-Fib Zr(B).

Figure 14 and Table 7 show the stability test of the most active catalyst studied: Mn-Fib Zr(B). Four consecutive runs showed almost no deactivation of the catalyst, shifting T_50_ values from 215 °C to 260 °C after the fourth evaluation. As can be observed, the catalyst partially deactivated during the second and third cycles, but from the fourth cycle onwards, the CO conversion profiles are identical. This would indicate the stabilization of the catalyst. Both the catalyst samples after one catalytic evaluation (one cycle) and the sample after being evaluated four times consecutively (four cycles) were characterized by XRD. As Figure S2 shows, the crystalline structure is maintained after one reaction cycle for all catalysts studied, whereas the cryptomelane signals appeared less intense in the case of the sample Mn-Fib Zr(B) after the stability tests. The slight deactivation could probably be due to this fact along with surface changes in the catalyst. This issue would require a detailed study, which exceeds the scope of this work and is proposed for future studies.

## 4. Conclusions

The use of the gliding arc plasma technique (glidarc) to deposit active phases in ceramic supports is a promising technique, and only a handful of works have been published on this issue. In this work, zirconia fibers were used as supports in order to deposit Mn and Fe oxides, with the aim of obtaining active catalysts for soot and CO oxidation reactions. On one hand, biomorphic zirconia fibers were synthesized utilizing cotton as a biotemplate (Zr(B)), and on the other hand, commercial zirconia fibers (Zr(C)) were employed for comparison. In the first case, it was noticed in SEM images that zirconia greatly maintained the morphology of cotton, which is probably a result of the chemical and thermal stability of this material. The tetragonal phase (t-ZrO_2_) was identified by XRD and LRS in the Fib Zr(B) sample, while bare commercial fibers (Fib Zr(C)) presented only the monoclinic phase (m-ZrO_2_).

Regarding the phases obtained from metal oxides deposited via glidarc plasma, the precipitate formed from the Mn precursor was clearly identified in Mn-Fib Zr(B) as cryptomelane (K_x_Mn_8_O_16_). Contrarily, for Mn-Fib Zr(C), the cryptomelane structure was not detected by XRD. On the other hand, for both Fe-containing samples, the hematite phase (α-Fe_2_O_3_) was evident in the diffractograms. In XPS analyses, the presence of potassium on the surface of Mn-supported samples was noticed, which is in line with the phase assignation performed previously (cryptomelane structure). Additionally, the average oxidation state (AOS) of Mn was calculated, and a similar value was obtained for both Mn samples (around 3.7); therefore it was assumed that both samples exhibit the same phase but with a slightly different Mn^3+^/Mn^4+^ ratio.

Bare zirconia fibers were almost inactive in both reactions tested (soot oxidation and CO oxidation), while the addition of iron produced no significant effects. However, given the more open structure of biomorphic fibers, while they show some small activity in soot conversion, bare commercial ones are totally inactive. In the case of CO oxidation, both types of fibers are inactive. The catalytic activity in both types of reactions evaluated improves when the cryptomelane structure (K_x_Mn_8_O_16_) is present, especially in the case of the solid–solid–gas reaction studied (soot oxidation). This improvement may be related to the mixed-valence states of Mn (Mn^3+^/Mn^4+^) along with the presence of K on the surface that favors soot-to-catalyst contact and the release of active oxygen species for soot oxidation. In the case of CO oxidation, the cryptomelane phase likely allows a fraction of CO molecules to enter into cryptomelane tunnels due to their low kinetic diameter (3.69 Å), thus favoring CO oxidation, given the higher residence time of CO molecules into the said tunnels. Moreover, consecutive CO oxidation runs showed the good stability of the Mn-Fib Zr(B) catalyst.

In view of the obtained results, future work will be performed to gain insight into the mechanism by which the glidarc technique proceeds, with the aim of optimizing the procedures employed, and also to gain insight into the reaction mechanisms of both reactions studied, mainly using Mn catalysts. Other catalytic applications are also foreseen, such as VOC oxidation reactions.

## Figures and Tables

**Table 1:** FTIR-ATR signal assignations for all catalysts.

Band Position [cm^−1^]	Assignment
3400, 1646	-OH, H-O-H (physiosorbed water)
1542, 1360, 1100	Adsorbed mono-dentate and bridged CO_3_^2−^
1140	Sulfate (SO_4_^2−^)
690, 436	Zr-O in t-ZrO_2_
727, 577, 490, 418	Zr-O in m-ZrO_2_
511	Mn-O in K_x_Mn_8_O_16_
530, 520, 434	Fe-O in α-Fe_2_O_3_
580	Fe-O in Fe_3_O_4_

**Table 2:** Mn/Zr and Fe/Zr atomic ratios from SEM—EDS (Figure 5) and ICP analyses.

Sample	M */Zr Atomic Ratios		M * Content (ICP, wt.%)
	Fiber Mapping (from Figure 5A) **	Big Mapping (from Figure 5B)	
***Mn-Fib Zr(B)***	0.05	0.18	4.0
***Fe-Fib Zr(B)***	0.73	0.65	5.4
***Mn-Fib Zr(C)***	0.08	0.20	3.8
***Fe-Fib Zr(C)***	0.36	0.35	4.8

* M = Mn or Fe. ** Average for three fibers analyzed per sample.

**Table 3:** Surface characteristics of Fe- or Mn-supported catalyst (XPS).

Sample	Binding Energy (eV)				O_ads_ (%)	Atomic Ratios		
	Zr 3d_5/2_	Zr 3d_3/2_	O_latt_	O_ads_		O_ads_/O_latt_	O_ads_/Zr	M */Zr
***Fib Zr(B)***	181.7	184.1	529.3	531.1	25.5	0.34	0.60	-
***Mn-Fib Zr(B)***	181.8	184.2	529.4	531.3	21.0	0.27	0.64	0.35
***Fe-Fib Zr(B)***	182.2	184.6	529.8	531.5	37.7	0.60	2.24	1.50
***Fib Zr(C)***	182.0	184.3	529.7	531.5	25.7	0.35	0.66	-
***Mn-Fib Zr(C)***	181.8	184.2	529.4	531.4	23.6	0.31	1.00	0.65
***Fe-Fib Zr(C)***	182.1	184.5	529.7	531.4	34.6	0.53	2.28	1.92

* M = Mn or Fe.

**Table 4:** Summary of T_50_, T_90_, and T_M_ values of Fib Zr(B) and Fib Zr(C) for both types of catalytic tests.

Catalyst	Soot Combustion *			CO Oxidation **	
	T_50_ [°C]	T_90_ [°C]	T_M_ [°C]	T_50_ [°C]	T_100_ [°C]
***Blank***	615	659	641	--	--
***Fib Zr(B)***	517	546	537	500	600
***Mn-Fib Zr(B)***	473	516	493	213	300
***Fe-Fib Zr(B)***	534	573	538	514	600
***Fib Zr(C)***	588	625	604	464	600
***Mn-Fib Zr(C)***	502	550	525	230	330
***Fe-Fib Zr(C)***	514	560	513	450	600

* Values obtained from Figure 12. ** Values obtained from Figure 13.

**Table 5:** A comparison of the activities of the best catalysts to those of other catalysts reported in the literature for soot combustion.

Catalysts [Reference]	Feed Composition	Soot–Catalyst wt. Ratio Type of Soot Type of Contact	T_M_ (°C) [T_50_ (°C)]
***Mn-Fib Zr(B)*** ***[this work]***	3.3% O_2_, 33% Ar, He balance	1:20 Printex U Wet contact	493 [473]
***Mn-Fib Zr(C)*** ***[this work]***	3.3% O_2_, 33% Ar, He balance	1:20 Printex U Wet contact	525 [502]
***Fe-Fib Zr(B)*** ***[this work]***	3.3% O_2_, 33% Ar, He balance	1:20 Printex U Wet contact	538 [534]
***Fe-Fib Zr(C)*** ***[this work]***	3.3% O_2_, 33% Ar, He balance	1:20 Printex U Wet contact	513 [514]
***CeO_2_ (fibers)*** ***[50]***	10% O_2_, N_2_ balance	1:9 Printex U Loose contact	553 [533]
***Mn_3_O_4_*** ***[51]***	0.05% NO, 5% O_2_, He balance	1:4 Printex U Loose contact	nr [510]
***MnOx-CeO_2_*** ***[52]***	0.1% NO, 10% O_2_, N_2_ balance	1:20 Real soot Loose contact	460 [nr]
***K/MnO_2_*** ***[53]***	10% O_2_, N_2_ balance	1:10 Printex U Loose contact	nr [478]

“nr” indicates that it is not reported.

**Table 6:** CO oxidation: An activity comparison of the best catalysts versus others reported in the literature.

Catalysts [References]	CO Oxidation	
	Feed Composition	T_50_ [°C]
***Mn-Fib Zr(B) [this work]***	1% CO, 2% O_2_, He balance	213
***Mn-Fib Zr(C)*** ***[this work]***	1% CO, 2% O_2_, He balance	230
***Mn-Fib Ce [13]***	1% CO, 2% O_2_, He balance	202
***Co-Fib Ce [54]***	1% CO, 2% O_2_, He balance	201
***Ce-Zr-O [55]***	0.1% CO, 10% O_2_, He balance	250
***MnOx-CeO_2_ [56]***	1.5% CO, 10% O_2_, N_2_ balance	225
***Cu-CeO_2_ [57]***	1.5% CO, 2.5% O_2_, N_2_ balance	250
***Ce-ZrO_2_ [58]***	1% CO, 4% O_2_, N_2_ balance	180

**Table 7:** CO oxidation: Stability runs.

*Catalyst*	*T*_50_ [°C]	*T*_100_ [°C]
***Mn-Fib Zr(B) cycle 1***	215	300
***Mn-Fib Zr(B) cycle 2***	241	343
***Mn-Fib Zr(B) cycle 3***	260	314
***Mn-Fib Zr(B) cycle 4***	260	314

## Data Availability

The original contributions presented in this study are included in the article. Further inquiries can be directed to the corresponding authors.

## Supplementary Materials

The following supporting information can be downloaded at https://www.mdpi.com/article/10.3390/ma18245479/s1, Figure S1: XPS spectra of O 1s region for catalysts supported on biomorphic fibers (a) and those supported on commercial fibers (b); Figure S2: XRD patterns of samples after CO oxidation evaluations. Catalysts supported on biomorphic ZrO_2_ fibers (a) and commercial ZrO_2_ fibers (b).
