# Supplementary material for: The Use of the Gliding Arc Plasma Technique to Deposit Fe or Mn Oxides on Fibrous Ceramic Supports for Reactions of Environmental Interest
Source: Materials (Basel). 2025 Dec 5;18(24):5479. doi: 10.3390/ma18245479 (PMC12734582; doi:10.3390/ma18245479)
Supplement: Supplementary file 1 [file materials-18-05479-s001.zip › materials-3981392-supplementary.pdf]

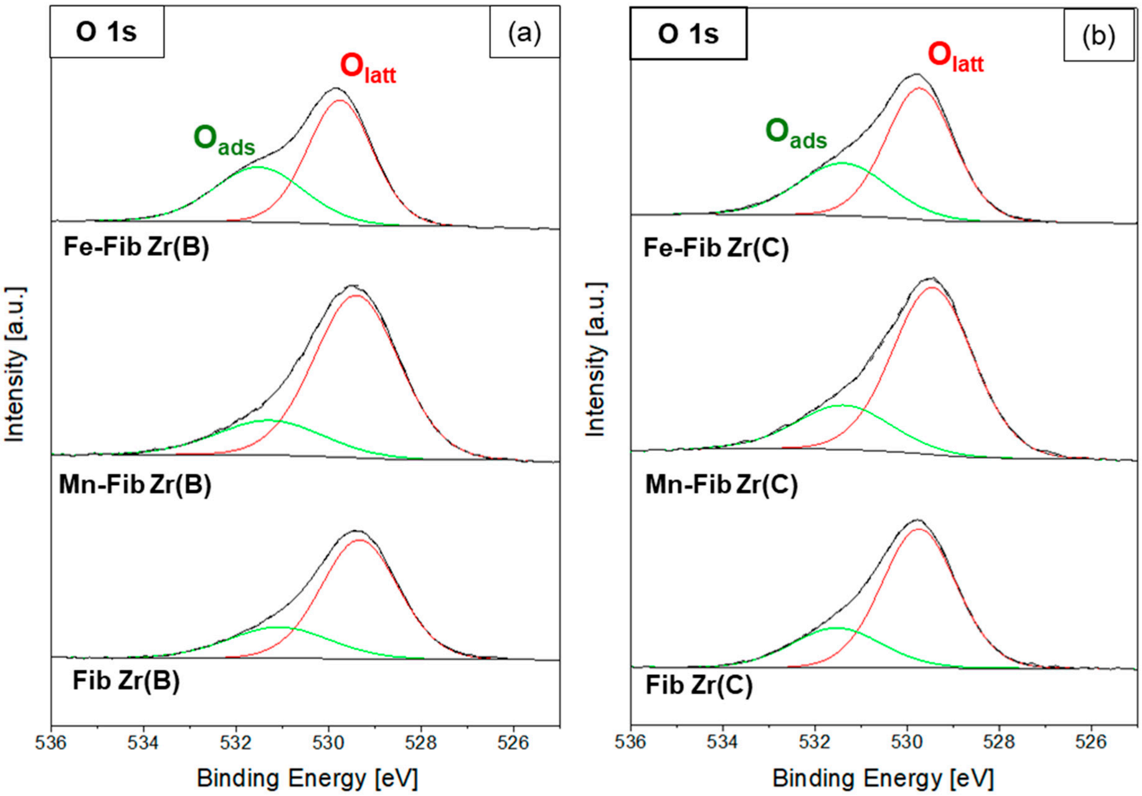

Figure S1. XPS spectra of O 1s region for catalysts supported on biomorphic fibers (a) and those supported on commercial fibers (b).

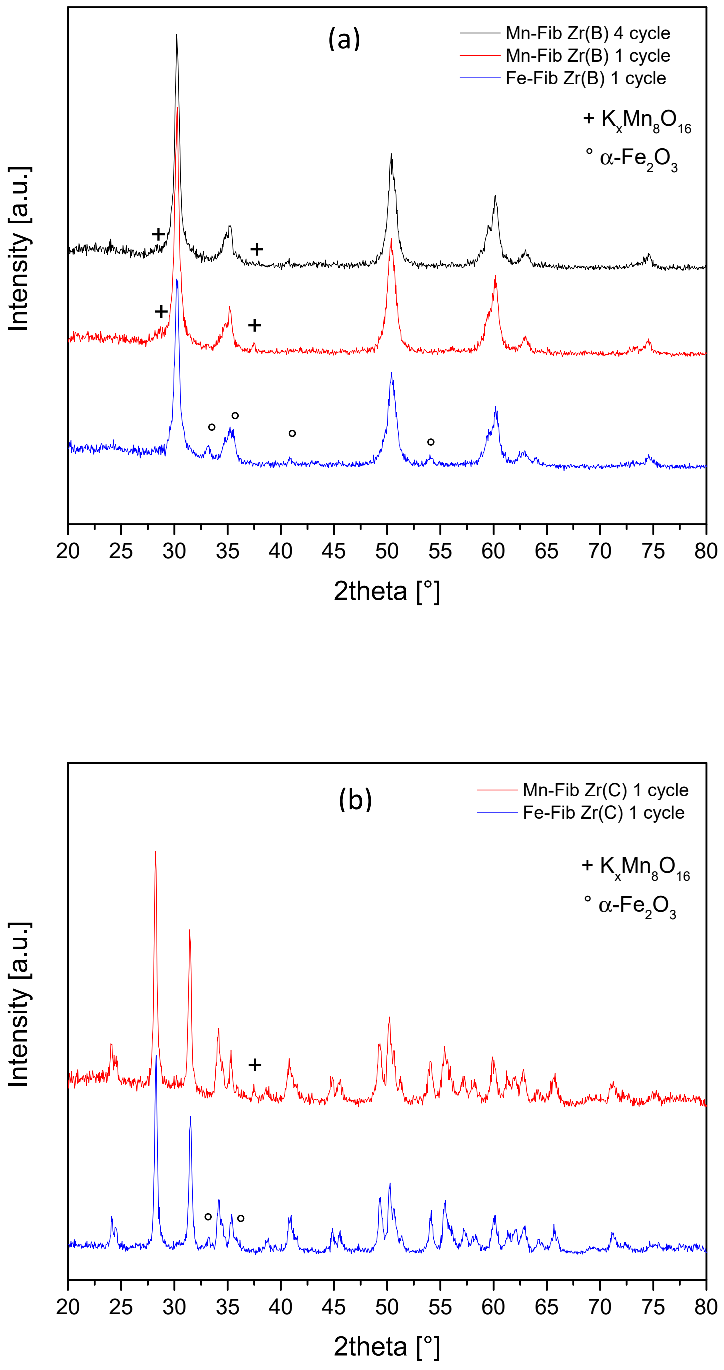

Figure S2. XRD patterns of samples after CO oxidation evaluations. Catalysts supported on biomorphic ZrO_2 fibers (a) and commercial ZrO_2 fibers (b).
